# Metabolome Profiling and Pathway Analysis in Metabolically Healthy and Unhealthy Obesity among Chinese Adolescents Aged 11–18 Years

**DOI:** 10.3390/metabo13050641

**Published:** 2023-05-08

**Authors:** Lingling Tong, Mei Tian, Xiaoyan Ma, Ling Bai, Jinyu Zhou, Wenqing Ding

**Affiliations:** 1 School of Public Health and Management, Ningxia Medical University, Yinchuan 750004, China; 2 Key Laboratory of Environmental Factors and Chronic Disease Control, Ningxia Medical University, Yinchuan 750004, China

**Keywords:** metabolites, pathways, metabolically healthy obesity (MHO), metabolically unhealthy obesity (MUO), children and adolescents

## Abstract

The underlying mechanisms of the development of unhealthy metabolic phenotypes in obese children and adolescents remain unclear. We aimed to screen the metabolomes of individuals with the unhealthy obesity phenotype and identify the potential metabolic pathways that could regulate various metabolic profiles of obesity in Chinese adolescents. A total of 127 adolescents aged 11–18 years old from China were investigated using a cross-sectional study. The participants were classified as having metabolically healthy obesity (MHO) or metabolically unhealthy obesity (MUO) based on the presence/absence of metabolic abnormalities defined by metabolic syndrome (MetS) and body mass index (BMI). Serum-based metabolomic profiling using gas chromatography–mass spectrometry (GC–MS) was undertaken on 67 MHO and 60 MUO individuals. ROC analyses showed that palmitic acid, stearic acid, and phosphate could predict MUO, and that glycolic acid, alanine, 3-hydroxypropionic acid, and 2-hydroxypentanoic acid could predict MHO (all *p* < 0.05) from selected samples. Five metabolites predicted MUO, 12 metabolites predicted MHO in boys, and only two metabolites predicted MUO in girls. Moreover, several metabolic pathways may be relevant in distinguishing the MHO and MUO groups, including the fatty acid biosynthesis, fatty acid elongation in mitochondria, propanoate metabolism, glyoxylate and dicarboxylate metabolism, and fatty acid metabolism pathways. Similar results were observed for boys except for phenylalanine, tyrosine and tryptophan biosynthesis, which had a high impact [0.098]. The identified metabolites and pathways could be efficacious for investigating the underlying mechanisms of the development of different metabolic phenotypes in obese Chinese adolescents.

## 1. Introduction

Metabolic disorders, especially obesity in children and adolescents, have increased in frequency and have become a major public health issue throughout the world [1]. Similarly, the standardized prevalence of obesity in Chinese children and adolescents has increased rapidly, from 1.7% in 1991–1995 to 9.6% in 2015–2019 [2].

Studies have shown that obesity can lead to metabolic abnormalities in the body, such as elevated blood pressure, type 2 diabetes, dyslipidemia, and metabolic syndrome [3]. Recent studies have found that obese individuals do not all have the same risk of developing metabolic derangements, and individuals in the same body mass index (BMI) category can also have different metabolic profiles. Some obese individuals, with so-called “metabolically healthy obesity (MHO)”, showed a more beneficial metabolic phenotype than metabolically unhealthy obese (MUO) individuals [4]. Keihani et al. found that individuals with the MUO phenotype showed higher risks of cardiovascular disease (CVD), type-2 diabetes, and all-cause mortality than those with the MHO phenotype [5]. Therefore, the timely classification and identification of obese individuals with different metabolic phenotypes and the early development of cost-effective individualized prevention and intervention programs are of great significance for reducing the risk of metabolic abnormalities in obese children and adolescents.

Currently, studies of the relevant factors and regulatory pathways for distinguishing the MHO and MUO phenotypes are still limited, and their relationships are unclear. Several studies have shown that diets, lifestyles, fat mass distributions, and other behavioral and environmental factors are associated with MHO status. For instance, in our previous study, lower arm fat mass amounts were found in the MHO group than in the MUO group among children and adolescents [6]. However, obesity and its related metabolic abnormalities result from a complex interaction between predisposing genetic and environmental factors. Therefore, these factors may not reveal the causes of the differences between the MHO and MUO phenotypes.

It is worth noting that metabolites, as intermediates or final products of different metabolic pathways, can reflect genetic and environmental exposures and their interactions. Metabolomics measures low-molecular-weight metabolites from biological samples and reveals the dynamic physiological changes that result from behavioral and clinical outcomes. Thus, metabolomics technologies are a more creative and comprehensive screening tool for exploring metabolites. A study of adults revealed key metabolomics profiles and pathways, such as fatty acid biosynthesis, that could distinguish the MHO and MUO phenotypes by using serum-based metabolomics technology [7].

Previous studies have applied metabolomics to investigate the metabolomic profiles and relevant metabolic pathways of obese children and adolescents [8,9]. However, few studies have used metabolomics to characterize the metabolic state of obese children and adolescents. Therefore, we aimed to identify metabolites to distinguish metabolically healthy from unhealthy obese Chinese children and adolescents in our study.

## 2. Methods

### 2.1. Study Population

**Figure 1:**
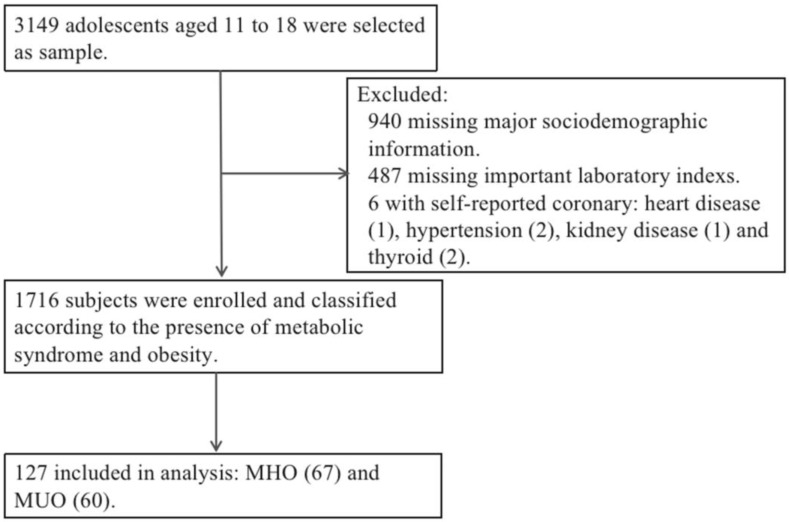
Participant screening and enrollment flow chart.

In this cross-sectional study, population-based sampling was used to select children and adolescents aged 11 to 18 years residing in Yinchuan, China, from October 2015 to November 2018. Six schools were randomly selected for different age subgroups. More than 100 children were sampled by a stratified cluster random sampling method per age group (each with half boys and half girls). Of those, 3149 subjects were selected, and 1673 subjects were excluded because they did not meet all the inclusion criteria or due to missing data. The remaining 1716 subjects were enrolled in the study and were classified according to their BMIs into two main groups: the MHO group (*n* = 67) and the MUO group (*n* = 60) (Figure 1). All participants completed a questionnaire, anthropometric measurements, blood sample collection, and clinical examination. We calculated the statistical power using G*Power software version 3.1.9.2 (G*Power Software Inc., Kiel, Germanyaccessed on) in a post hoc manner, as follows: power(1 − β) = 0.80, (α = 0.05, effect size = 0.5, n1 = 67, n2 = 60). The exclusion criteria included subjects with diabetes mellitus, hypertension, and renal, thyroid or heart diseases. The study was conducted according to the standards of the Declaration of Helsinki and approved by the Ethics Committee of Ningxia Medical University (2021-G053). All methods were performed in accordance with the relevant guidelines and regulations. Informed consent was provided by all participants and their parents/guardians.

### 2.2. Anthropometric and Biochemical Analysis

Anthropometric and biochemical data were collected by trained staff using a standardized procedure. Weight, height and waist circumference (WC) were all measured accurately twice to the nearest 0.1 kg and 0.1 cm while the subjects were barefoot and wearing lightweight clothing. The mean values were used to calculate BMIs (calculated as weight per height2 (kg·m^−2^)).

Systolic and diastolic blood pressures (SBP and DBP) were measured by automatic electronic sphygmomanometers (OMRON HEM-7012; Omron, Kyoto, Japan). These blood pressure measurements were obtained three times for all individuals at 1–2 min intervals, and an appropriate cuff was selected according to the midpoint of the right circumference. The mean values of the last two measurements were recorded as the BP values.

Venous blood samples were collected from all subjects by direct venipuncture after a 12-h overnight fast. Total cholesterol (TC), high-density lipoprotein (HDL-C), triglyceride (TG), low-density lipoprotein (LCL-C) and fasting plasma glucose (glucose oxidase method) levels were directly measured with a 7060C automatic chemistry analyzer (Hitachi, Tokyo, Japan) and were tested by enzymatic methods.

### 2.3. Definitions

According to the Working Group on Obesity (WGOC) in China, obese children and adolescents were defined by BMI values [10]. Metabolic syndrome (MetS) components were used to define metabolic abnormalities [11] as follows: (1) WC ≥ 90th percentile for age and sex [12]; (2) SBP and/or DBP ≥ 90th percentile for age, sex and height [13]; (3) TG levels ≥ 1.24 mmol·L^−1^; (4) low serum HDL-C ≤ 1.03 mmol·L^−1^; and (5) fasting glucose ≥ 5.6 mmol·L^−1^. Thus, the criterion for MHO were defined as obese with 0–1 abnormalities, and for MUO it was defined as obese with ≥2 abnormalities.

### 2.4. Metabolome Profile

Plasma samples were collected and stored at −80 °C. The samples were thawed for 30 min at 4 °C. Fifty microliters of a sample was taken and placed in a 1.5 mL EP tube. Next, 5 μL of adonitol (Sigma–Aldrich, St. Louis, MO, USA) was added after adding 200 µL of the methanol (CNW Technologies, Duesseldorf, Germany) extract. The mixture was vortexed for 30 s, sonicated for 10 min in an ice water bath, and then centrifuged at 12,000 rpm for 15 min at 4 °C. A total of 180 µL of supernatant was pipetted into a 1.5 mL EP tube, and 20 μL of each sample to be tested was taken and mixed as a QC (quality control) sample. The metabolites were dried in a vacuum concentrator before being mixed with 80 μL of methoxamine salt reagent (e.g., 20 mg/mL methoxamine hydrochloride (TCI, Tokyo, Japan) dissolved in pyridine (Adamas, Shanghai, China)). The mixture was gently mixed and incubated at 80 °C for 30 min. After that, 100 μL of BSTFA (containing 1% TMCS, *v*/*v*) (REGIS Technologies, Morton Grove, IL, USA) was added to each sample, and the mixture was incubated at 70 °C for 1.5 h. The mixture was then cooled to room temperature, and 5 μL of FAMEs (Dr Ehrenstorfer GmbH, Augsburg, Germany) (dissolved in chloroform (Adamas, Shanghai, China)) was added. The machine was tested in a random order.

The metabolomics profile analysis was performed by a gas chromatography mass spectrometer (GC-MS) (GC-MS-QP2010, Shimadzu, Japan). Samples were analyzed by applying the following instrument parameters. Chromatographic separations were performed on an Agilent DB-5MS (30 m × 250 μm × 0.25 μm) column. A volume of 1 µL of derivatized sample solution was injected through a split/splitless injector operating at a temperature of 280 °C, at a split ratio of 20:1, and with a helium carrier gas flow rate of 1 mL·min^−1^ in constant flow mode. Gas saver flow (25 mL·min^−1^) was switched on 15 s after sample injection. The temperature program began at 70 °C with a hold time of 4 min, followed by a linear temperature ramp of 8 °C per min up to 300 °C, followed by a hold time of 10 min. The oven temperature was then allowed to cool to 70 °C before the next injection. The transfer line temperature was held at 250 °C. The mass spectrometer source was operated at a temperature of 200 °C in EI mode, with an electron energy of 70 eV. Data were acquired over the range of 35-800 *m*/*z* and at an acquisition rate of 20 Hz.

### 2.5. Statistical Analyses

The original data were obtained from 127 experimental samples, from which 2169 peaks were extracted, and 2168 peaks were retained after preprocessing. All data were normalized to an internal standard (e.g., heptadecanoic acid). Principal component analysis (PCA), as an unsupervised analysis that reduces the dimensionality of the data, was carried out to visualize the distribution and grouping of the samples. The 95% confidence interval in the PCA score plot was used as the threshold to identify potential outliers in the dataset. Partial least square discriminant analysis (PLS-DA) and orthogonal partial least square discriminant analysis (OPLS-DA) established the supervised model to identify those variables with significance, and it is also important to distinguish the MHO from the MUO groups. Metabolite differences were tested by t-tests. The VIP > 1 and *p* < 0.05 variables contributed greatly to the model, and the data were analyzed with SIMCA Statistics software version 14.0 (Umetrics, Malmo, Sweden). Logistic regression was used to track the optimal metabolic markers with *p* values of less than 0.05. To further identify the metabolites, we performed ROC curve analyses. All relevant detected metabolites were mapped to metabolic pathways using the Kyoto Encyclopedia of Genes and Genomes (KEGG) database, and 80 human metabolism pathways were selected for further analysis [14]. To clarify the metabolic pathways of differential metabolites, a metabolic pathway analysis was performed with the MetaboAnalyst 3.0 tool (available online at http://www.metaboanalyst.ca/MetaboAnalyst/ (accessed on 10 July 2020)). We performed two different types of pathway analyses, including enrichment and topology analyses. The sex and BMI variables were considered as potential confounding factors and were controlled in the analysis.

## 3. Results

### 3.1. Demographic and Clinical Characteristics of Study Participants

The demographic and biomedical data are reported in Table 1. A total of 127 adolescents with obesity were included. The MHO and MUO groups had similar sexes and ages (*p* > 0.05). Individuals with MHO had significantly lower BMI, SBP, DBP, LDL, TC, and TG values than those with MUO (*p* < 0.05).

### 3.2. Serum Metabolic Profiles of MHO and MUO

**Figure 2:**
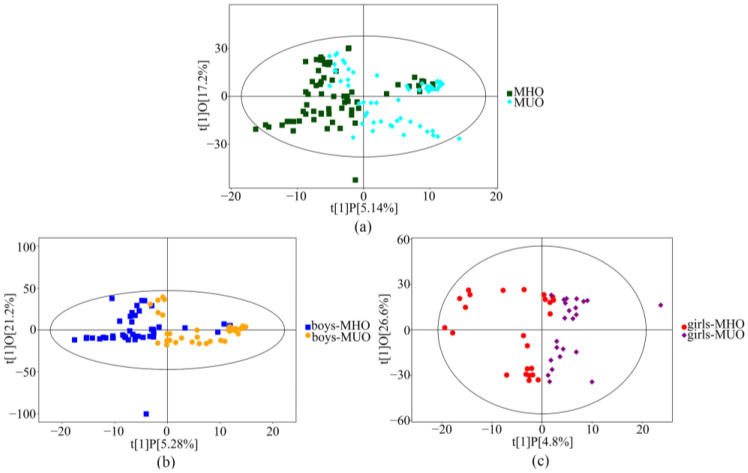
The scatter plot of PLS-DA to distinguish the MHO from MUO. The PLS-DA was conducted based on detectable metabolites. ((**a**): Total, 127; (**b**): Boys, 80; (**c**): Girls, 47).

A principal component analysis showed that nearly all samples were within the 95% confidence interval. The OPLS-DA plots showed a clear separation of serum metabolites between the MHO and MUO groups in the total subjects (Figure 2a). The serum metabolic profiles of the MHO and MUO subjects among boys (Figure 2b) and girls (Figure 2c) had similar results to all subjects.

### 3.3. Differential Metabolites

For all subjects, 20 metabolites with VIP > 0.5 and *p* < 0.05 were considered differentially expressed between the MHO and MUO groups. We also found differentially expressed metabolites for six (girls) and for 25 (boys) between the MHO and MUO. In all subjects, six metabolites increased and 14 metabolites decreased in the MUO group compared to the MHO group. Among girls, four metabolites decreased and two metabolites increased. Out of 25 metabolites, 17 increased and eight decreased in the MUO group compared to the MHO group in boys. (Table 2).

### 3.4. Metabolite Pathways of Differential Metabolites

**Figure 3:**
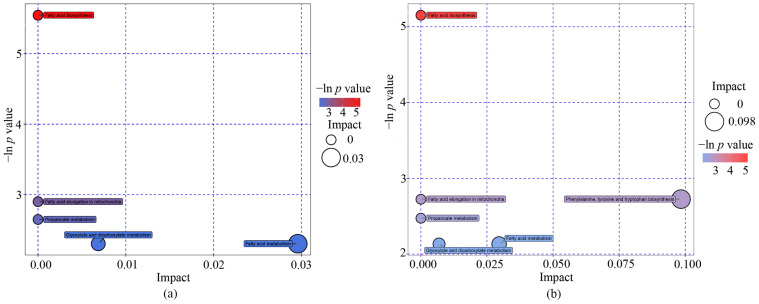
The pathway impact in topology analyses and *p* values in the enrichment analyses conducted by metabolomics pathway analysis. A total of twenty top metabolites for all the participants (**a**) and twenty-five top metabolites for boys (**b**) were involved in the pathway analysis. The size of pathway symbols represents significant levels of enrichment analysis, and the color of the pathway symbols represents the impact factors.

The metabolite pathways of different metabolites are shown in Table 3. Fatty acid biosynthesis pathways were significantly enriched (*p* < 0.05), and four pathways had low *p* values (*p* < 0.1), including the fatty acid elongation in mitochondria, propanoate metabolism, glyoxylate and dicarboxylate metabolism, and fatty acid metabolism pathways. Furthermore, two pathways, including glyoxylate and dicarboxylate metabolism and fatty acid metabolism obtained from topology analysis, had high pathway impacts greater than 0.001 (Table 3 and Figure 3a). Similar results were shown among boys, except for phenylalanine, tyrosine and tryptophan biosynthesis, which had a high impact [0.098] (Table 3 and Figure 3b).

### 3.5. Further Identification of Metabolites

Multivariable logistic regression revealed that the MUO phenotype was associated with nine metabolites in all participants, and included glycolic acid, palmitic acid, stearic acid, phosphate, asparagine, alanine, 3-hydroxypropionic acid, 2-hydroxypentanoic acid, and isoleucine (all *p* < 0.05). In girls, the MUO phenotype was associated with two metabolites: glycyl-proline and glucosamine. An MUO status was associated with 19 metabolites, including 2-hydroxybutanoic acid, 2-hydroxypentanoic acid, 3-hydroxypropionic acid, 5-methyluridine, acetophenone, alanine, asparagine, beta-gentiobiose, cyanoalanine, furoylglycine, galactinol, glycerol-alpha-phosphate, glycolic acid, isocitric acid minor, isoleucine, palmitic acid, salicylaldehyde, shikimic acid, and stearic acid (Table 4). The AUCs for these metabolites are presented in Tables S1 and S2. The AUCs for palmitic acid, stearic acid, and phosphate for predicting MUO and glycolic acid, alanine, 3-hydroxypropionic acid, and 2-hydroxypentanoic acid for predicting MHO were all greater than 0.6 (all *p* < 0.05) in the total population. In boys, five metabolites could predict MUO, 12 metabolites could predict MHO, and only two metabolites from girls could predict MUO.

## 4. Discussion

In this study, we evaluated metabolome profiling which differentiated the MHO and MUO phenotypes, and several potential metabolic pathways, which modulated the different metabolic profiles of obesity among Chinese adolescents. A panel of metabolites, including palmitic acid, stearic acid, and 2-hydroxybutanoic acid, among others, was chosen to accurately differentiate between subjects with the MHO and MUO. The main metabolic pathways associated with the MUO phenotypes included fatty acid biosynthesis, fatty acid elongation in mitochondria, propanoate metabolism, glyoxylate and dicarboxylate metabolism, fatty acid metabolism and phenylalanine, tyrosine and tryptophan biosynthesis (only in boys).

In this study, we found that stearic acid and palmitic acid might help to initially identify MUO phenotypes in Chinese adolescents independent of age, sex, and BMI. Palmitic acid and stearic acid are the most abundant saturated fatty acids (SFAs) in the diet [15]. In a similar vein, some studies have also shown that patients with type 2 diabetes or hyperlipidemia and mice fed high-fat diets exhibited high plasma SFA levels, including the two acids mentioned above [16,17,18]. Furthermore, some studies have also shown that palmitic acid and stearic acid are more toxic to pancreatic β-cells, resulting in altered pancreatic β-cell function [18,19]. In addition, both can induce inflammation and endoplasmic reticulum stress in in vitro studies and animal studies [20,21]. Thus, these two fatty acids helped us to distinguish obese adolescents who were metabolically unhealthy.

This KEGG ID (C00249) refers to the metabolite palmitic acid, which was involved in both fatty acid elongation in the mitochondria and fatty acid metabolism. The latter had a higher topological analysis impact factor in this study. The pathways mentioned above are related to mitochondrial function: ATP production from mitochondrial β-oxidation depends largely on the catabolism of saturated fatty acids containing 16 carbons and, to a lesser extent, those with 18 carbons such as palmitate and stearate [22]. The synthesis of long-chain saturated fatty acids involves the malonyl-CoA-dependent elongation of fatty acids. An animal study showed that when palmitate was perfused into rat hearts, some of the perfused palmitates underwent a chain extension to form stearate [22]. Stearic acid and palmitic acid also played key roles in the fatty acid biosynthetic pathway, which was identified in our pathway analysis. They elaborated that the elongation of fatty acid chains as described above is localized on the outer surface of the mitochondrial membrane. Malonyl-CoA, the ultimate two-carbon donor for chain elongation, which is present on the outer mitochondrial membrane, is synthesized with mitochondrial β-oxidation-derived acetyl-CoA, and provides malonyl-CoA for fatty acid chain lengthening, which can inhibit the excessive oxidation of mitochondrial fatty acids. In addition, malonyl-CoA induces adipogenesis and stimulates glucose oxidation in response to insulin-activated acetyl-CoA carboxylase. Therefore, these pathway abnormalities may reflect abnormal mitochondrial function in obese children and adolescents. This finding supports the hypothesis proposed by Welsh et al. that SFA is a significant contributor to β-cell dysfunction [23].

Moreover, this KEGG ID (C05984) refers to the metabolite 2-hydroxybutanoate, which was found to identify MHO phenotypes for Chinese adolescents varying in age, sex, and BMI in our study. Another study showed that 2-hydroxybutyric acid participates in the synthesis of substrate branched chain amino acids (BCAAs) [24]. This study suggested that the BCAA and 2-hydroxybutyric concentrations during fasting predicted insulin resistance (IR) in adolescents, which could prevent other diseases in adulthood. 2-hydroxybutyric acid is associated with impaired β-cell function in the human body, and has been recognized as an early indicator of IR and type 2 diabetes in recent years [25]. 2-hydroxybutyric acid was also involved in the propanoate metabolism pathway in this study. Impaired metabolism of the propionic acid pathway not only leads to propionic acidemia but can also induce abnormal blood glucose [26]. When metabolic abnormalities caused by insulin resistance or hyperglycemia occur in obese children and adolescents, more pyruvate and acetyl-CoA are produced by more glucose through the glycolytic pathway, which leads to oxidative stress. To overcome oxidative stress, hepatocytes produce glutathione via cysteine anabolic metabolism, which produces the byproduct α-ketobutyrate, which is then converted to 2-hydroxybutyric acid [25]. In addition, M.Schwab et al. found that the intermediate of propionate metabolism, propionyl-CoA, has a toxic effect on mitochondrial function by inhibiting pyruvate oxidation [27]. These findings suggest that metabolites and related pathways potentially play a role in regulating the metabolic state of early-life obesity through mitochondria.

This KEGG ID (C00160) refers to the metabolite glycolic acid, which was a differential metabolite with *p* < 0.05 in different metabolic phenotypes and was involved in the glyoxylate and dicarboxylic acid metabolic pathways in this study. Glycolic acid is required for oxalic acid production, which has been found to increase the production of reactive oxygen species from mitochondria [28]. Furthermore, we found that isocitric acid, an intermediate of the tricarboxylic acid cycle, could also identify the MHO phenotype in obese children and adolescents, and it was found to be positively correlated with BMI, abdominal obesity, HOMA-IR, and triglycerides (TG) by Jennifer E et al. [29]. These studies combined suggest that the different metabolic phenotypes of obese children and adolescents are related not only to mitochondrial dysfunction but also to energy metabolic homeostasis.

In our pathway analysis of boys, phenylalanine, tyrosine and tryptophan biosynthesis (C00493) scored highest for their effects (0.098). An animal study showed that apolipoprotein-I (a major component of high-density lipoprotein) knockout mice exhibited abnormalities in the phenylalanine, tyrosine and tryptophan biosynthesis metabolic pathways [30]. Phenylalanine is converted to tyrosine by phenylalanine hydroxylase. This conversion stimulated the synthesis of catecholamines in the brain, which resulted in a decrease in blood pressure [31]. Shikimic acid has been identified as a significant metabolite for distinguishing MHO (Table S2), and was involved in the pathways mentioned above. Shikimic acid is a phenolic compound and an intermediate metabolite in tryptophan synthesis. A small proportion of tryptophan is catalyzed by tryptophan hydroxylase to form 5-hydroxytryptamine, a vasoconstrictor in peripheral tissues. Patients with coronary artery disease have reduced plasma tryptophan concentrations and increased kynurenine/tryptophan levels due to the conversion of the majority of tryptophan to kynurenine [31]. Therefore, the metabolites and pathways may be associated with lipid and blood pressure abnormalities, which are important for the identification and development of different metabolic phenotypes in obese children and adolescents. Consequently, these findings suggest that 2-hydroxybutyric acid, glycolic acid, shikimic acid, alanine, and other metabolites associated with the MHO phenotype indicate that the MHO phenotype has an early-stage cardiovascular disease risk profile.

Notably, our study found that only glycyl-proline and glucosamine were associated with the MUO phenotype in girls, and no relevant metabolic pathways were identified, which suggested that there were sex differences in the metabolites and pathways in identifying the MUO and MHO phenotypes. Several studies based on metabolic disorders and cardiovascular disease have also found that females typically have more beneficial metabolic profiles and lower risks of cardiovascular disease [32,33]. Similarly, a cohort study showed that transitional MHO females had healthier metabolic profiles than their male counterparts. This could explain the sex differences. In addition, the relationship of glycyl-proline and glucosamine with the MUO phenotype has not been studied. Proline is a protective substance for membranes and enzymes in living organisms, and is a free radical scavenger that protects the vascular endothelium from damage [34]. Its plasma levels are often altered by endovascular lesions, but the role of glycyl-proline in cardiovascular metabolism is not yet clear.

To the best of our knowledge, few studies have previously assessed untargeted metabolomics in Chinese children and adolescents with different metabolic phenotypes. However, several limitations of our study should be noted here. First, in a cross-sectional study, we failed to demonstrate the causal association between the identified metabolic pathways or metabolites and different metabolic phenotypes in obese children and adolescents, and more prospective studies are needed for validation. Second, some interpretations may be inaccurate, although previous studies of animals or adults can explain the biological rationality of metabolites found due to the species, age differences, and limited research related to children and adolescents in metabolites. Third, it is noteworthy that the sample size is relatively small. Fourth, in untargeted analyses, it is possible that one peak may correspond to multiple metabolites with similar mass over charge values, leading to potentially less accurate results in pathway analysis. Some metabolites may not be identified in humans and need to be examined manually, even in pathway analysis and with the KEGG dataset. Fifth, we did not adjust for puberty-related variables due to a lack of diet and physical activity data in our study, which may have affected the results of the study. Finally, the definition of MHO may lead to results that are inconsistent with other studies, and different metabolic abnormalities may have different underlying causes.

## 5. Conclusions

In conclusion, we have successfully identified several related metabolites that distinguish the MUO group from the MHO group, including palmitic acid, stearic acid, glycolic acid, 2-hydroxybutanoic acid, shikimic acid, and others. These metabolites are involved in several important metabolic pathways, including fatty acid biosynthesis, fatty acid elongation in mitochondria, propanoate metabolism, glyoxylate and dicarboxylate metabolism, fatty acid metabolism, and phenylalanine, tyrosine and tryptophan biosynthesis (only in boys). Future studies are needed to validate our findings; that is, abnormal mitochondrial function may be associated with different metabolic states in obese children and adolescents, and more studies are needed to investigate the potential mechanisms of metabolic dysregulation in obesity.

## Figures and Tables

**Table 1:** Demographic and clinical characteristics in MHO and MUO participants.

Variables	MHO (*n* = 67)	MUO (*n* = 60)	*p* Value
Age (years)	14.15 ± 1.66	14.73 ± 1.67	0.051
Female, n (%)	24 (35.8)	23 (38.3)	0.700
Height (cm)	168.9 ± 8.7	170.7 ± 8.6	0.259
Weight (kg)	80.6 ± 11.9	86.5 ± 13.7	0.010
BMI (kg/m^2^)	28.1 ± 2.5	29.6 ± 3.21	0.005
WC (cm)	91.8 ± 8.1	93.6 ± 10.2	0.335
SBP (mmHg)	119 ± 12	124 ± 12	0.013
DBP (mmHg)	69 ± 7	74 ± 7	<0.001
TC (mmol/L)	3.68 ± 0.73	4.29 ± 0.79	<0.001
TG (mmol/L) *	1.10 (0.92,1.32)	2.13 (1.67,2.62)	<0.001
HDL-C (mmol/L)	1.24 ± 0.19	1.05 ± 0.21	<0.001
LDL-C (mmol/L)	2.02 ± 0.66	2.47 ± 0.84	0.001
Fasting glucose (mmol/L)	4.77 ± 0.36	4.66 ± 0.40	0.173

Normally distributed data are expressed as the mean ± SD. The median and interquartile range was used for skewed variables; Abbreviations: MHO, metabolica healthy obesity; MUO, metabolic unhealthy obesity; BMI, body mass index; WC, waist circumstance; SBP, systolic blood pressure; DBP, diastolic blood pressure; TC, total cholesterol; TG, triglyceride; HDL-C, high-density lipoprotein cholesterol; LDL-C, low-density lipopretein cholesterol; *: Skewed distributions were logarithmically transformed for the statistical test.

**Table 2:** Differential metabolites between the MHO and MUO groups.

Metabolite	Mean Relative Quantitative Value in MUO Group	Mean Relative Quantitative Value in MHO Group	VIP	*p* Value	Fold Change
Total					
glycolic acid	0.022	0.013	1.243	0.010	1.671
palmitic acid	1.293	1.668	2.804	0.002	0.775
glucose	0.078	0.056	1.735	0.035	1.391
stearic acid	1.243	1.582	2.667	0.003	0.786
2-hydroxybutanoic acid	0.054	0.030	1.383	0.006	1.081
galactose	0.394	0.317	1.087	0.038	1.242
phosphate	0.443	0.681	1.949	0.010	0.651
asparagine	0.035	0.017	1.399	0.003	1.503
alanine	0.139	0.075	1.878	0.011	1.858
glutamate	0.008	0.013	2.117	0.034	0.609
cyanoalanine	0.030	0.020	2.433	0.029	1.510
3-hydroxypropionic acid	0.167	0.102	1.266	0.015	1.639
2-hydroxypentanoic acid	0.025	0.012	1.411	0.004	1.997
hippuric acid	0.067	0.039	1.521	0.020	1.715
acetophenone	0.076	0.046	1.877	0.015	1.663
beta-gentiobiose	0.029	0.018	0.854	0.022	1.605
maltotriose	0.006	0.004	0.843	0.021	1.540
isoleucine	0.031	0.019	1.187	0.021	1.645
myristyl myristate	0.000	0.001	1.511	0.046	0.710
salicylaldehyde	0.000	0.001	1.371	0.009	0.770
Girls					
benzylalcohol	0.001	0.000	0.686	0.040	2.407
2-ketoadipic acid	0.000	0.000	1.074	0.028	1.819
glycyl-proline	0.000	0.000	3.020	0.009	0.582
glucosamine	0.000	0.000	2.555	0.007	0.552
pyrophosphate	0.000	0.000	1.100	0.042	0.575
2-ketobutyric acid	0.000	0.000	3.156	0.015	0.583
Boys					
2-hydroxy-2-methylbutanoic acid	0.021	0.035	1.867	0.036	0.594
2-hydroxybutanoic acid	0.064	0.034	1.053	0.016	1.869
2-hydroxypentanoic acid	0.030	0.014	0.902	0.006	2.183
3-hydroxypropionic acid	0.198	0.114	1.283	0.024	1.743
5-methyluridine	0.035	0.024	1.171	0.006	1.452
acetophenone	0.090	0.052	1.638	0.021	1.746
alanine	0.167	0.088	1.482	0.023	1.898
asparagine	0.045	0.021	2.004	0.003	2.105
beta-gentiobiose	0.036	0.019	0.603	0.011	1.860
cyanoalanine	0.039	0.025	3.286	0.017	1.602
furoylglycine	0.000	0.000	0.981	0.036	0.822
galactinol	0.047	0.030	0.904	0.020	1.538
galactose	0.400	0.306	1.044	0.039	1.307
glutamate	0.008	0.015	1.649	0.024	0.549
glycerol-alpha-phosphate	0.017	0.026	0.895	0.010	0.681
glycolic acid	0.026	0.015	2.050	0.027	1.674
isocitric acid	0.090	0.066	1.218	0.025	1.351
isogluconic acid	0.113	0.065	1.230	0.031	1.745
isoleucine	0.041	0.024	1.079	0.025	1.701
maltotriose	0.007	0.004	1.479	0.029	1.631
palmitic acid	1.205	1.667	2.854	0.002	0.723
phosphate	0.433	0.690	1.675	0.032	0.628
salicylaldehyde	0.000	0.001	1.633	0.003	0.731
shikimic acid	0.050	0.028	0.807	0.013	1.816
stearic acid	1.155	1.555	2.705	0.003	0.743

**Table 3:** Metabolite pathways of differential metabolites.

Pathway Name	Total Number of Compounds in the Pathways	Enrichment Analysis *p* Value	Topology Analysis Impact Factor	Name of Metabolites and KEGG ID
Total (N = 127)				
Fatty acid biosynthesis	49	0.004	0.000	Stearic acid cpd:C01530; Palmitic acid cpd:C00249
Fatty acid elongation in mitochondria	27	0.055	0.000	Palmitic acid cpd:C00249
Propanoate metabolism	35	0.071	0.000	2-Hydroxybutyric acid cpd:C05984
Glyoxylate and dicarboxylate metabolism	50	0.099	0.007	Glycolic acid cpd:C00160
Fatty acid metabolism	50	0.099	0.030	Palmitic acid cpd:C00249
Boys (n = 80)				
Fatty acid biosynthesis	49	0.006	0.000	Stearic acid cpd:C01530;Palmitic acid cpd:C00249
Fatty acid elongation in mitochondria	27	0.066	0.000	Palmitic acid cpd:C00249
Propanoate metabolism	35	0.084	0.000	2-Hydroxybutyric acid cpd:C05984
Glyoxylate and dicarboxylate metabolism	50	0.118	0.007	Glycolic acid cpd:C00160
Fatty acid metabolism	50	0.118	0.030	Palmitic acid cpd:C00249
Phenylalanine, tyrosine and tryptophan biosynthesis	27	0.067	0.098	Shikimic acid cpd:C00493

**Table 4:** Results of significant metabolites using logistic regression analysis.

Metabolite	β	S.E.	Wald	*p* Value
Total ^a^				
glycolic acid	−27.820	12.448	4.995	0.025
palmitic acid	0.856	0.360	5.657	0.017
glucose	−7.386	3.997	3.145	0.065
stearic acid	0.834	0.372	5.024	0.025
2-hydroxybutanoic acid	9.024	7.527	1.438	0.231
galactose	−1.380	1.123	1.509	0.219
phosphate	0.761	0.379	4.026	0.045
asparagine	−15.913	6.284	6.412	0.011
alanine	−3.000	1.418	4.475	0.034
glutamate	22.905	16.295	1.976	0.160
cyanoalanine	−15.101	7.701	3.845	0.050
3-hydroxypropionic acid	−3.230	1.409	5.259	0.022
2-hydroxypentanoic acid	−9.604	4.186	5.263	0.022
hippuric acid	−5.234	3.003	3.037	0.081
acetophenone	−5.504	2.883	3.646	0.056
beta-gentiobiose	−14.408	7.883	3.340	0.068
maltotriose	−89.458	48.507	3.401	0.065
isoleucine	−16.832	8.126	4.291	0.038
myristyl myristate	576.787	485.312	1.412	0.235
salicylaldehyde	1354.748	794.353	2.909	0.088
Girls ^b^				
benzylalcohol	−1090.490	657.516	2.751	0.097
2-ketoadipic acid	9133.505	4866.245	3.523	0.061
glycyl-proline	4800.051	2407.659	3.975	0.046
glucosamine	5353.060	2541.747	4.435	0.035
pyrophosphate	4161.921	2452.639	2.944	0.086
2-ketobutyric acid	9133.505	4866.245	3.523	0.061
Boys ^b^				
2-hydroxy-2-methylbutanoic acid	14.974	8.681	2.975	0.085
2-hydroxybutanoic acid	−10.303	4.774	4.657	0.031
2-hydroxypentanoic acid	−25.500	10.365	6.053	0.014
3-hydroxypropionic acid	−3.964	1.716	5.337	0.021
5-methyluridine	−47.939	18.506	6.711	0.010
acetophenone	−7.110	3.528	4.061	0.044
alanine	−3.398	1.698	4.004	0.045
asparagine	−19.827	7.431	7.119	0.008
beta-gentiobiose	−19.175	9.517	4.060	0.044
cyanoalanine	−20.389	9.125	4.992	0.025
furoylglycine	2916.777	1479.492	3.887	0.049
galactinol	−19.112	8.661	4.869	0.027
galactose	−2.548	1.552	2.694	0.101
glutamate minor	38.914	20,759	3.514	0.061
glycerol-alpha-phosphate	38.874	17.372	5.007	0.025
glycolic acid	−32.656	15.120	4.664	0.031
isocitric acid minor	−12.577	6.127	4.152	0.042
isogluconic acid	−4.772	2.591	3.391	0.066
isoleucine	−18.384	9.066	4.112	0.043
maltotriose	−111.404	59.697	3.483	0.062
palmitic acid	1.408	0.534	6.952	0.008
phosphate	0.868	0.466	3.476	0.062
salicylaldehyde	2711.686	1206.927	5.048	0.025
shikimic acid	−14.479	7.244	3.995	0.046
stearic acid	1.404	0.566	6.163	0.013

^a^ Model adjusted for age, sex and BMI; ^b^ Model adjusted for age and BMI.

## Data Availability

The data presented in this study are available in the article and in the Supplementary Material.

## Supplementary Materials

The following supporting information can be downloaded at: https://www.mdpi.com/article/10.3390/metabo13050641/s1, Table S1: ROC curve analysis of metabolites for predicting metabolically unhealthy obesity. Table S2: ROC curve analysis of metabolites for predicting metabolically healthy obesity.
