# Supplementary material for: Metabolome Profiling and Pathway Analysis in Metabolically Healthy and Unhealthy Obesity among Chinese Adolescents Aged 11–18 Years
Source: Metabolites. 2023 May 8;13(5):641. doi: 10.3390/metabo13050641 (PMC10221088; doi:10.3390/metabo13050641)
Supplement: Supplementary file 1 [file metabolites-13-00641-s001.zip › metabolites-2351218-supplementary.pdf]

Table S1 ROC curve analysis of metabolites for predicting metabolically unhealthy obesity

Test variables	AUC	95% CI	*p* value
Total			
glycolic acid	0.376	0.278-0.473	0.016
palmitic acid	0.620	0.521-0.719	0.020
stearic acid	0.627	0.528-0.725	0.014
phosphate	0.622	0.520-0.724	0.018
asparagine	0.407	0.307-0.508	0.072
alanine	0.372	0.274-0.470	0.013
3-hydroxypropionic acid	0.393	0.294-0.491	0.037
2-hydroxypentanoic acid	0.356	0.258-0.454	0.005
isoleucine	0.415	0.316-0.515	0.100
Girls			
glycyl-proline	0.732	0.587-0.877	0.006
glucosamine	0.739	0.596-0.882	0.005
Boys			
2-hydroxybutanoic acid	0.351	0.226-0.475	0.022
2-hydroxypentanoic acid	0.343	0.219-0.467	0.016
3-hydroxypropionic acid	0.363	0.240-0.485	0.035
5-methyluridine	0.312	0.194-0.431	0.004
acetophenone	0.336	0.214-0.459	0.012
alanine	0.344	0.220-0.469	0.017
asparagine	0.385	0.257-0.514	0.078
beta-gentiobiose	0.365	0.243-0.487	0.038
cyanoalanine	0.336	0.212-0.459	0.012
furoylglycine	0.638	0.514-0.762	0.034
galactinol	0.336	0.217-0.456	0.012
glycerol-alpha-phosphate	0.671	0.552-0.790	0.009
glycolic acid	0.360	0.236-0.484	0.032
isocitric acid minor	0.297	0.180-0.414	0.002
isoleucine	0.376	0.252-0.499	0.057
palmitic acid	0.661	0.538-0.784	0.013
salicylaldehyde	0.693	0.576-0.810	0.003
shikimic acid	0.334	0.215-0.453	0.011
stearic acid	0.659	0.535-0.784	0.014

Table S2 ROC curve analysis of metabolites for predicting metabolically healthy obesity

Test variables	AUC	95% CI	*p* value
Total			
glycolic acid	0.624	0.527-0.722	0.016
palmitic acid	0.380	0.281-0.479	0.020
stearic acid	0.373	0.275-0.472	0.014
phosphate	0.378	0.276-0.480	0.018
asparagine	0.593	0.492-0.693	0.072

alanine	0.628	0.530-0.726	0.013
3-hydroxypropionic acid	0.607	0.509-0.706	0.037
2-hydroxypentanoic acid	0.644	0.546-0.742	0.005
isoleucine	0.585	0.485-0.684	0.100
girls			
glycyl-proline	0.268	0.123-0.413	0.006
glucosamine	0.261	0.118-0.404	0.005
boys			
2-hydroxybutanoic acid	0.649	0.525-0.774	0.022
2-hydroxypentanoic acid	0.657	0.533-0.781	0.016
3-hydroxypropionic acid	0.637	0.515-0.760	0.035
5-methyluridine	0.688	0.569-0.806	0.004
acetophenone	0.664	0.541-0.786	0.012
alanine	0.656	0.531-0.780	0.017
asparagine	0.615	0.486-0.743	0.078
beta-gentiobiose	0.635	0.513-0.757	0.038
cyanoalanine	0.664	0.541-0.788	0.012
furoylglycine	0.362	0.238-0.486	0.034
galactinol	0.664	0.544-0.783	0.012
glycerol-alpha-phosphate	0.329	0.210-0.448	0.009
glycolic acid	0.640	0.516-0.764	0.032
isocitric acid minor	0.703	0.586-0.820	0.002
isoleucine	0.624	0.501-0.748	0.057
palmitic acid	0.339	0.216-0.462	0.013
salicylaldehyde	0.307	0.190-0.424	0.003
shikimic acid	0.666	0.547-0.785	0.011
stearic acid	0.341	0.216-0.465	0.014
